# Conceptual Modelling of Two Large-Scale Mine Water Geothermal Energy Schemes: Felling, Gateshead, UK

**DOI:** 10.3390/ijerph19031643

**Published:** 2022-01-31

**Authors:** David Banks, Jonathan Steven, Adam Black, John Naismith

**Affiliations:** 1 Holymoor Consultancy Ltd., 360 Ashgate Road, Chesterfield S40 4BW, Derbyshire, UK; 2 Groundwater & Geothermal Services Ltd., Low Cross Buildings, 4 Low Cross Street, Brampton CA8 1NP, Cumbria, UK; jsteven@ggs-geothermal.co.uk; 3 Lanchester Wine Cellars Ltd., Greencroft Estate, Tower Road, Annfield Plain, Stanley DH9 7XP, County Durham, UK; adam.black@lanchesterwines.co.uk; 4 TownRock Energy Ltd., East Woodlands House, Dyce AB21 0HD, Aberdeen, UK; john@townrockenergy.com

**Keywords:** geothermal, mine water, heat pump, hydrogeology, coal mine

## Abstract

A conceptual model is presented of two MW-scale low enthalpy mine water geothermal heat pump schemes that are being developed in Tyneside, UK. The Abbotsford Road scheme (54.955° N 1.556° W) is operating (as of May 2021) at 20–30 L/s, abstracting groundwater (and heat) from an unmined Coal Measures Upper Aquifer System (UAS) and reinjecting to the deeper High Main Aquifer System (HMAS), associated with the High Main (E) coal workings and the overlying High Main Post sandstone. A similar scheme, 700 m away at Nest Road (54.959° N 1.564° W), abstracts at 40 L/s from the HMAS, recovers heat from the mine water and reinjects the thermally spent water to deeper workings associated with the Hutton (L), Harvey-Beaumont (N) (and possibly other) coal seams, termed the Deep Mined Aquifer System (DMAS). The three aquifer systems are vertically discontinuous and possess different hydraulic (storage, transmissivity and continuity) properties that would have been near-impossible to predict in advance of drilling. At the sites, 10 boreholes were drilled to obtain five usable production/reinjection boreholes. Development of mine water geothermal energy schemes thus carries a significant project risk, and also a potential ongoing maintenance burden related to iron hydroxide scaling. These do not preclude mine water geothermal as a useful low carbon heating and cooling technology, but the involvement of skilled hydrogeologists, hydrochemists, mining and groundwater engineers is a pre-requisite.

## 1. Introduction

**Figure 1:**
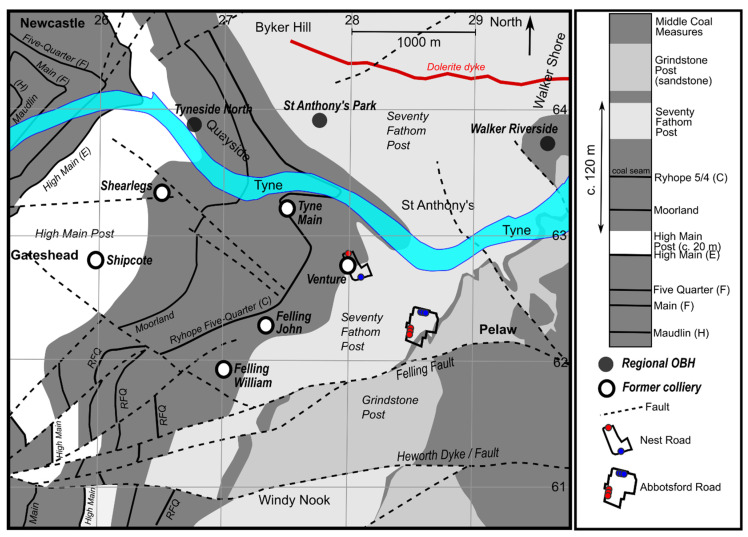
Simplified geological map of the Felling area, showing regional Observation Boreholes (OBH). Geological information derived from British Geological Survey (https://www.bgs.ac.uk/, accessed on 25 November 2021) mapping. Contains Open Geoscience public sector information licensed under the Open Government Licence v3.0.

**Figure 2:**
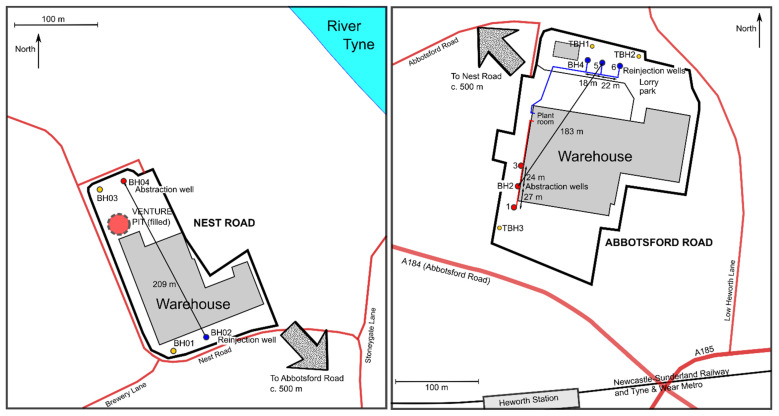
Schematic maps of the (**left**) Nest Road and (**right**) Abbotsford Road sites.

The abstraction-reinjection borehole systems at Nest Road (54.959° N 1.564° W) and Abbotsford Road (54.955° N 1.556° W), Gateshead, are the first large-scale examples of Mine water Geothermal Energy Schemes (MGES) operating in the UK (Figure 1 and Figure 2). Such MGES are a widely discussed, but relatively seldom implemented, means of delivering low-carbon space-heating and cooling [1,2]. They typically use mine water pumped from flooded mine voids, which is then passed through a heat exchanger, coupled to a heat pump. The heat pump/exchanger system extracts heat from the mine water (typically ΔT = 3–5 °C of temperature drop) and the chilled, thermally spent water is discharged, either to the environment or back to the mine workings. The rate of heat extracted (H_ex_) from the mine water is given by:

H_ex_ = Q × ρ.c × ΔT     (in kW_th_) (1)

where Q = mine water flow rate (L/s)

- ρ.c is the volumetric heat capacity of water (=c. 4.19 kJ/L/K)
- ρ = water density (kg/L)
- c = specific heat capacity of water (kJ/kg/K)
- ΔT = temperature change across heat exchanger (K)

The rate of heat supplied (H_sup_) to the customer by the heat pump is given, in slightly simplified form, by:

H_sup_ = H_ex_/(1 − 1/COP)     (in kW_th_) (2)

where COP is the coefficient of performance of the heat pump. The quantity of electrical energy (E) used by the heat pump is given by

E = H_sup_/COP     (in kW_e_) (3)

In the UK, MGES technologies have hitherto only been implemented on a relatively small scale at schemes such as Lumphinnans and Shettleston, Scotland (both c. 65 kW; now no longer operating [3,4]), Crynant, Wales (35 kW; [5,6]), Dawdon, Co. Durham, England (12 kW; [7]) and Markham, Bolsover, England (20 kW; [8]).

Significantly larger schemes are now planned at Seaham Garden Village (Dawdon, Co. Durham [9,10]), Gateshead Baltic (Tyne and Wear [11]), Hebburn (South Tyneside [12]) and Caerau (Wales [13]). At the time of writing, however, the two schemes developed at Lanchester Wines’ warehouse facilities at Abbotsford Road and Nest Road, both in Felling, Gateshead, are by far the largest MGES operating in the UK (Table 1). This paper concisely describes the schemes and the current conceptual model of the site (which is continuously evolving). It also touches on some of the development and operational challenges that have been encountered.

The site at Abbotsford Road is installed with 2.4 MW of heat pump capacity. It contains three abstraction wells drilled to depths of 110–123 m, three reinjection wells drilled to depths of c. 155 m and three observation wells. Water is pumped at up to around 30 L/s through a plate heat exchanger, coupled to the heat pumps (although the site is licensed for 49 L/s). Applying equations (1) to (3) and assuming ΔT = 4 °C and COP = 4, it is estimated that over 100 L/s water might be required to service the full 2.4 MW capacity. The site is thus under development to attempt to increase the yield.

The site at Nest Road is located c. 700 m to the north-west of Abbotsford Road and installed with 1.2 MW of heat pump capacity. It contains one abstraction well (BH04) drilled to 131 m, one reinjection well (BH02) drilled to 280 m and two observation wells. Applying Equations (1)–(3) and assuming ΔT = 4 °C and COP = 4, it is estimated that over 50 L/s is required to service the 1.2 MW capacity. Water can be abstracted from BH04 at c. 13 °C at rates of 60–70 L/s, but the reinjection borehole BH02 has, at the time of writing, only been tested at up to 40 L/s. As of May 2021, the site is operating (in the form of extended operational testing) under a limited duration licence from the Environment Agency (EA).

A large amount of hydrogeological and thermal data has been collected from the sites, manually, using borehole-specific sensors and using the sites’ Building Management Systems (BMS). The data presented here do not represent the full available data set. We have selected key periods of data, water analyses and pumping tests which we believe are key to informing the conceptual model.

At Abbotsford Road, because the site is only capable of delivering c. 30 L/s of groundwater, plans for further development are shortly to be set in motion, which will involve deepening selected unproductive boreholes to deeper horizons and/or levels of working, and/or drilling of additional boreholes. Operational testing and reporting to the Environment Agency are ongoing to secure long-term abstraction licensing at Nest Road. While these works and testing may have been partially completed by the date of publication, this paper summarises the history and conceptual understanding of the sites as of May 2021. As the paper describes an evolving, real-life case study, rather than academic research, the paper follows a non-standard structure: it first presents the hydrology, geology and mining geometry (Section 2 and Section 3), before discussing each site in turn and the interaction between them (Section 4, Section 5 and Section 6), and finally drawing the findings together in a conceptual model and conclusions (Section 7 and Section 8).

## 2. The Sites: Topography, Hydrology and Geology

The Nest Road and Abbotsford Road sites are separated by c. 700 m (Figure 1 and Figure 2). Both lie around 300 m south of the River Tyne estuary in the Felling area of Gateshead, about 3.7 km ESE of the city centre of Newcastle-upon-Tyne.

The River Tyne itself has a tidal range of over 4 m near its mouth into the North Sea at North Shields, c. 11 km downstream from Felling (55.007° N 1.440° W [14]). The tidal influence in the Tyne extends to around 31 km upstream from its mouth. There is typically a chemical stratification in the Tyne Estuary, with fresh water flowing downstream above a basal layer of saline water and an intermediate mixing zone [15]. At Tynemouth, the annual average air temperature is 9.4 °C and the annual precipitation is 597 mm [16]. At Howdon (c. 54.99° N 1.50° W), the surface temperature in the Tyne was around 11.5 °C, increasing to c. 12.7 °C at the bed, in October 1960 [15].

The Quaternary geology of the sites is very variable; the Nest Road site lies close to the axis of a major buried valley, probably representing a Devensian subglacial meltwater channel, typically filled by tills, sands, silts and laminated clays [17], with a floor potentially as deep as −18 m asl [18]. At Nest Road, the Quaternary superficial sequence is dominated by buried channel fills (late Devensian glaciolacustrine laminated clay and silt, with lenses of sand and occasionally gravel) with possible boulder clay at depth. As early as 1817, it was noted [19] that such channel fills often contained “quick” sands, necessitating complex iron casing operations when sinking shafts through them. At Abbotsford Road, south of the main buried channel axis, the superficial deposits are thinner and likely to comprise a greater component of glaciofluvial/glacial deposits.

In terms of pre-Quaternary geology, both sites are located on, or very close to, the subcrop of the Seventy Fathom Post sandstone of the Westphalian (Upper Carboniferous) Middle Coal Measures sequence [18]. The sequence comprises alternating mudstones, siltstones, sandstones and minor coals before reaching the first extensively worked coal seam, the High Main or ‘E’ seam at over 100 m depth. The High Main seam is typically overlain by another major sandstone, the High Main Post, of c. 20 m thickness [20]. The regional dip is eastward, such that the High Main seam and High Main Post outcrop around 2 km west of the Lanchester Wines sites (Figure 1). The sandstones or “posts” above the High Main (E) seam of the Coal Measures sequence are known for providing high yields of groundwater; the Seventy Fathom Post was reported in 1864 [21] as yielding flows of 114 to 152 L/s (presumably recorded while sinking mine shafts).

Beneath the High Main (E) seam, the Coal Measures sequence continues, as illustrated by the sequence recorded from Felling Colliery’s Venture Pit (Table 2).

## 3. Felling Colliery

When Lanchester Wines required a low-carbon source of heating for its warehouses, the most attractive resource appeared to be the groundwater contained within the Coal Measures rocks beneath the sites, and within the flooded mine workings of Felling Colliery. This was one of numerous, interlinked collieries developed in Gateshead (on the south bank of the Tyne) and Newcastle (on the north). This section will describe the mining geometry and history in some detail, because the mode of working, and the connectivity between voids of adjacent collieries and seams strongly control the hydraulic behaviour of the mine water.

The Tyneside area is one of the oldest coal mining districts in the United Kingdom; its mining history and geology are summarised in considerable detail by, for example [19,20,23,24,25]. Although coal was worked in the Felling area since at least the 17th Century, Felling (or Brandling Main) colliery was founded in 1779 and worked through to the early 1930s.

**Figure 3:**
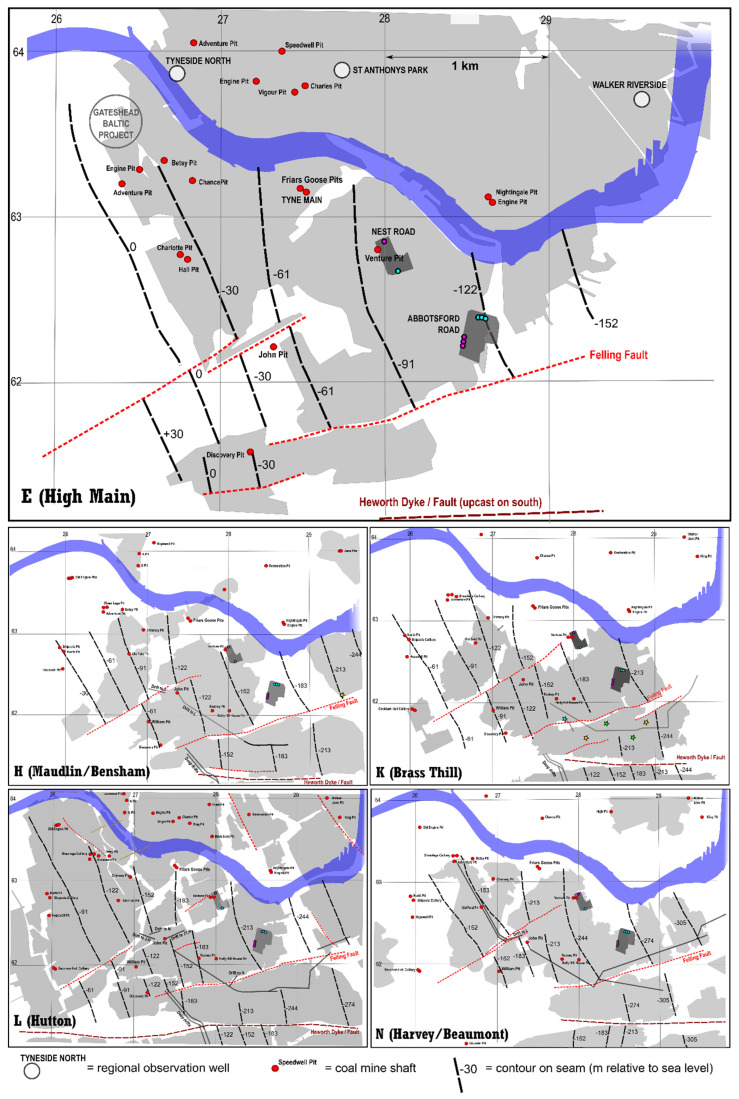
Schematic maps of Tyneside with footprints of workings, major coal pits, faults, structure contours and regional Observation Boreholes (OBH) indicated.

Felling Colliery incorporated workings of different ages in multiple coal seams (Figure 3, Table 2); these seams are designated by letters of the alphabet as well as seam names, due to some confusion over seam name duplication. The worked seams were predominantly the High Main (E), Maudlin-Bensham (H), Six-Quarter (J), Brass Thill (K, although this seam was not worked below the Nest Road site), Hutton (L) and Harvey-Beaumont (N) seams. There were likely also limited late workings in the deep Busty (Q) or Brockwell (S) seams [26]. Felling Colliery was broadly bounded by:

- the Tyne Estuary in the north.
- the Heworth Fault/Dyke in the south, which has a downthrow to the north of c. 25 fathoms (46 m [19]) and to the south of which were the workings of Heworth Colliery.
- Tyne Main/Old Fold collieries to the west.
- Wardley Colliery in the east.

Felling was accessed by a number of shafts, the most important being the John and William shafts (Figure 1). Another shaft, the 3 m diameter Venture Pit (located within the Nest Road site), was sunk, prior to the 19th Century, to the High Main (E) seam, then deepened to the Hutton (L) seam in 1801. It was abandoned, with the rest of Felling Colliery, in the early 1930s [27] and was subsequently filled and then pressure grouted in 1983 using 755 tonnes cement grout [22].

The High Main (E) coal was the most important seam in the Tyneside area; almost always more than 1.5 m thick, it was worked intensively throughout the entire area [20]. By 1811, the High Main workings at Felling were essentially worked out and the focus of extraction shifted to the “Low Main” (probably referring to the Brass Thill (K) or Hutton (L)) seam). By 1812, the 212 m deep William Pit was the main upcast (air furnace) shaft, with the 187 m deep John Pit acting as the downcast shaft. On 25 May 1812, a series of underground (methane) explosions took place around the John and William shafts, resulting in the loss of 92 lives [28].

In 1842, workings in the High Main (E) seam were abandoned and “tubbed off” at Tyne Main colliery. At the time [26], “an arrangement was entered into with the owners of Felling, Walker, Wallsend, Willington and Heaton Collieries, under which they contributed to the cost of keeping the large pumping engine at Friar’s Goose Pit at work to prevent the water from passing to the dip”. Friars Goose shaft, at Tyne Main colliery, thus became one of the main loci of regional dewatering—the above statement illustrating that it was hydraulically connected, at least at the level of the High Main (E) seam, to Felling in the east and Walker to the north. By 1849, Friar’s Goose was pumping 1170 gallons per minute (89 L/s) of mine water. The water pumped was saline and formed the basis of a number of alkali works in the area [29,30,31,32,33], producing sodium sulphate (“saltcake”) and thereafter sodium carbonate. Brine springs were also recorded at several other locations nearby, including Walker and Birtley Collieries [19].

The High Main (E) workings of Felling Colliery are linked westward to those of Tyne Main and Friars Goose, which in turn are widely regionally interconnected and extend below the Tyne Estuary to those of Walker Colliery on the north shore of the Tyne (Figure 3). The High Main (E) workings are not, however, extensively or deliberately connected to deeper levels of working at Felling, unless the connections are via old, unfilled and unsealed shafts. As at Tyne Main, it is likely that abandoned High Main (E) workings would have been “tubbed out” on abandonment to prevent shallow mine water progressing downward to deeper workings, and allowing the High Main mine water to be handled by Friar’s Goose pumping station.

In contrast, workings in the deeper H to N seams are better connected vertically via a number of staple shafts and drifts. There are limited potential roadway/drift hydraulic connections from these deeper seams to the west (Abandonment Plans Plan 10456, 10540 and 11125 Part 2, held by the Coal Authority): (a) from the Hutton (L) near John pit westwards towards the Maudlin (H) workings of Old Fold/Tyne Main collieries, (b) from the Brass Thill (K) near John Pit westwards towards the Old Fold/Tyne Main collieries, with connections up to the Six Quarter (J) seam, (c) in the Harvey Beaumont (N) seam westwards towards the Old Fold/Tyne Main collieries.

There are also drift connections (d) south from Felling Brockwell (S) to Heworth Colliery’s Three-Quarters (R) and Brockwell (S) workings and (e) east from the Maudlin (H), Brass Thill (K) and Beaumont (N) workings of Felling Colliery to the Brass Thill (K), Beaumont (N) and/or Hutton (L) seams of Wardley Colliery (abandonment plans D1117/D1036 (45-2861 and 45-2862) and 11125 pts. 2 and 4, held by the Coal Authority). These southward and eastward drifts are all reported to be sealed with water dams, however.

## 4. Abbotsford Road

The Abbotsford Road site is a c. 4½ Ha plot just north of the A184 Abbotsford Road and Heworth railway station in Felling Gateshead (54.955° N 1.556° W), occupied by Lanchester Wines warehouse facility and a neighbouring bakery depot. It is located at an elevation of between 34–36 m asl (injection boreholes, in the north) to 38–43 m asl (abstraction boreholes, in the south), around 300 m south of a bend in the River Tyne estuary.

### 4.1. Boreholes

Three 6″ diameter investigation wells were drilled in 2015, and subsequently retained as observation wells, as follows:

- TBH1 was drilled to 102 m, in the area of the reinjection wells, plain cased to c. 53 m, then open hole.
- TBH2 was drilled to 152 m, in the area of the reinjection wells. It encountered the High Main (E) seam at the base of the borehole, with tentative indications of working. The borehole was lined with plain casing to 76 m, below which was slotted casing to 124 m then open hole
- TBH3 was drilled to c. 102 m, immediately south of abstraction BH1, plain cased to c. 53 m, then open hole.

Six full-scale production boreholes were then completed in early 2016, as follows. Boreholes BH1, 2 and 3 were drilled towards the south-western side of the Abbotsford Road site to depths of 117, 110 and 123 m respectively. These were the intended abstraction wells and were all constructed similarly. A large diameter borehole was initially drilled to c. 20 m and 508 mm steel casing set and grouted. Drilling then continued at 450 mm to total depth, and a string of 315 mm OD/285 mm ID PVC casing/screen inserted (ungrouted) from surface to near the base of the hole. The PVC string was plain casing to 17–21 m depth and thereafter slotted with 4 mm slot size. 7–10 m Drift were encountered in BH2 and TBH3, typically comprising Made Ground, over loose sandy soil over sandy boulder clay. Coal was encountered at c. 73 m bgl, representing the unworked Threequarters (C) seam; the High Main (E) was not reached. The strata were alternating mudstones, siltstones and sandstones, but with a high proportion of sandstones below c. 55 m depth.

Boreholes BH4, 5 and 6 were drilled towards the north of the site to depths of 156, 155 and 155 m respectively. Construction was similar to the abstraction boreholes, with 508 mm steel casing being installed and grouted to c. 20 m, thereafter drilled at 450 mm. BH4 and BH6 were left open hole below 20 m, while in BH5, a 315/285 mm PVC casing string was inserted to the base of the borehole, with the section 18.1 m bgl to base having 4 mm aperture slots. Coal and broken ground were encountered in BH4 at 152.5 to 156 m, and coal was encountered in BH5 and BH6 at the base of the borehole, representing the worked High Main (E) seam. The High Main Post sandstone could not be unequivocally identified in any of the reinjection wells, except for BH5, where a thick sandstone was logged from 110 m to at least 136 m bgl. 155 mm ID uPVC injection main was installed in BH4 to BH6 to a depth of 60 m bgl. Around the reinjection well sites, 7–12 m Quaternary superficial deposits were encountered in TBH1 and TBH2, typically comprising Made Ground over stiff boulder clay.

Rest water levels at the site are around +3 to +5 m asl.

### 4.2. Initial Test Pumping

Step testing in early 2016 suggested that BH1, 2 and 3 had yields of 30 L/s each, while BH4, 5 and 6 had yields of 8–20 L/s [34]. These yields were not sustained during subsequent combined testing, however, most likely due to the very close proximity (25–27 m spacing at surface) and consequent interference between the wells. Verticality surveys indicate that a number of boreholes are off-vertical, so spacing at abstraction/discharge depths may differ by several metres from the surface spacing.

Initial combined abstraction-reinjection test pumping was carried out in June 2016, which indicated that a combined abstraction—reinjection rate of 42–43 L/s could be sustained for 48 h (5.9 L/s from BH1 with 21.5 m, drawdown, 23.6 L/s from BH2 with 18 m drawdown, 13.9 L/s from BH3 with 23 m drawdown), with the rate being increased to 52 L/s for 2 h. For the reinjection boreholes, the yields and upconings were reported as 15.4 L/s/12.4 m in BH4, 16.9 L/s/10.8 m in BH5 and 10.0 L/s/16.5 m in BH6. This initial testing established that BH2 had a significantly higher specific capacity during combined operation (113 m^2^/d) than the other boreholes, and that BH4 and BH5 were the two most efficient reinjection wells (107 and 135 m^2^/d).

Further extended test pumping was carried out between 1 December 2017 and 30 December 2017 [35], with BH1, 2 and 3 pumping intermittently at combined rates of 30-35 L/s, with pumping almost continuous between 14 and 26 December (12 days). Rest water levels in BH1, 2 and 3 were around +3 m asl. The response in BH1, BH2 and BH3 was quasi-Theisian, with equilibrium not being achieved during the shorter pumping periods but approximate equilibrium being attained after around 3 days into the 12-day pumping period, with drawdowns of 13.5 m, 17 m and 27 m being attained in BH1, 2 and 3 respectively. Unfortunately, no test records document how the total flow of 30 L/s was divided between the boreholes, although temperature data suggest that the yield being derived from BH1 was negligible. Abstraction water temperatures of 12–13 °C were measured in BH2 and BH3.

Rest water levels in BH4, 5 and 6 were similar to the abstraction boreholes. The reinjection boreholes showed rather different responses to testing in December 2017, with a lower rate of change of water level than the pumping boreholes. In particular, BH6 exhibited a non-Theisian, quasi-linear, response. Convincing equilibrium was not achieved during the 12-day period of near continuous pumping. Total upconing reached 28 m in BH4 (to +31 m asl), 16 m in BH5 and 17 m in BH6. The reinjection flow was distributed between the three boreholes according to settings of gate valves at the borehole heads (set to achieve manageable upconing in each hole). It is reported [35] that “most of the recharge water went into BH4 with less flow being directed into BH5 and a much smaller flow into BH6”.

A full Heat Access Agreement (CA11/MHR/0015/N) was issued to Lanchester Wines by the Coal Authority on 25 May 2017 (notwithstanding the fact that the groundwater—and thus the heat—is being abstracted from an unmined Coal Measures aquifer, rather than from mine workings, with the cooled reinjected water being returned to the level of the worked High Main (E) seam). A full abstraction licence was granted by the Environment Agency (NE/023/0006/023) on 17 July 2018 permitting the extraction of 49 L/s (instantaneous and daily average) and 882,000 m^3^/y (average 28 L/s, or 49 L/s for 208 full heating days per year).

At the time (2018) when the current authors became involved as main consultants to Lanchester Wines, only BH2 was yielding significant quantities of water of up to 30 L/s, while BH1 and BH3 were discontinued from operation (BH3 was not significantly used following November 2018, due to excessive drawdown). BH4 and BH5 were accepting the vast majority of the reinjection flow. The system has continued to provide space heating for Lanchester Wines’ warehouse to date, although the flows are less than required to support the full design heating load. At the time of writing, plans are underway to deepen BH1 to the High Main (E) seam and BH6 to the Brass Thill (K) or Hutton (L) seam, with a view to increasing the potential abstraction and rejection capacity.

A large quantity of monitoring data has been recovered from the site, but this paper has been very selective in reproducing data that shed light on the conceptual model of the system, and that illustrate some of the challenges it has faced. This paper reports results only up until a cut-off date of May 2021. The results of the planned deeper drilling at Abbotsford Road are thus excluded from this paper, but will no doubt be reported when testing is completed and data interpreted.

### 4.3. Operational Monitoring

**Figure 4:**
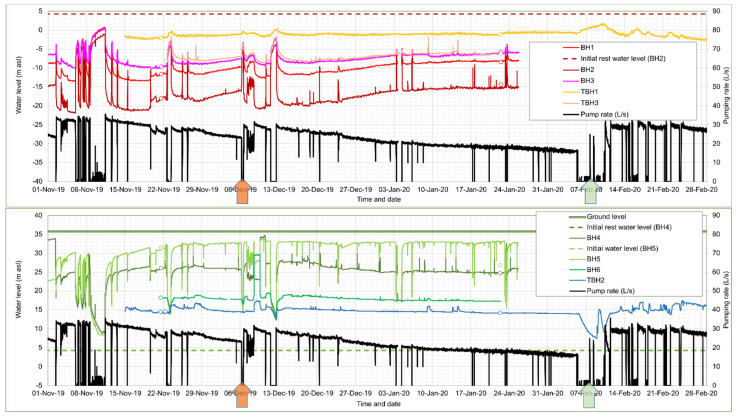
Operational monitoring of the Abbotsford Road site from November 2019 to February 2020. The arrows represent citric acid flushing of the plate heat exchanger of 6 December 2019 and oxalic acid cleaning and rehabilitation of BH2 around 7 February 2020. Small circles represent manual dips to calibrate automatically logged data. During the period, only BH2 was operating as an abstraction well, and only BH4 and BH5 as reinjection wells.

Selected operational groundwater monitoring data are presented in Figure 4.

**Figure 5:**
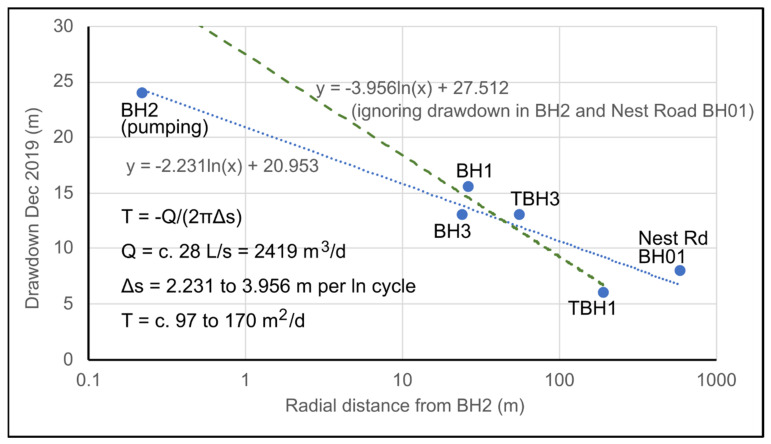
Drawdown versus log distance from BH2 for abstraction during December 2019, when a yield of 28 L/s is assumed. A drawdown at Nest Road BH01 of at least 8 m is assumed (RWL = +3–4 m asl and dynamic water level = −4 to −5 m asl, see Figure 9).

The data illustrate the behaviour of the wells during the period November 2019 to February 2020. During this period, BH2 was operating almost continuously, albeit with a slowly declining production rate. Short periods of inactivity can be seen, during which water levels recover in a broadly Theis-like manner. The operational drawdown in BH2 is around 24 to 25 m for a yield of 28–30 L/s, and drawdown declines with distance from the borehole in a log-linear manner. The drawdowns versus distance from BH2 for December 2019 are shown in Figure 5, which broadly suggest a transmissivity for the aquifer penetrated by BH2 of 100–170 m^2^/d, which is remarkably high for a Coal Measures sandstone.

It is especially noteworthy that borehole TBH1, despite being located close to the reinjection wells, experiences several metres drawdown in groundwater head. This suggests that vertical connectivity between aquifer horizons is very poor and that BH1, 2 and 3, TBH3 and TBH1 (all of which are 100–123 m deep), reflect groundwater heads in an upper aquifer, disconnected from deeper strata. Moreover, it also suggests that the reinjection wells are almost exclusively returning water to strata deeper than 110 m depth (i.e., the High Main Post and High Main (E) workings). If reinjected water were entering at a higher level, one would expect to see a reinjection response in TBH1.

The 152 m deep TBH2 does respond to reinjection, in the opposite manner to TBH1, as does the largely unused reinjection well BH6. The two utilised reinjection wells BH4 and BH5 achieve groundwater heads of up to +33 and +28 m asl (within c. 3 and 8 m of the surface). BH5 has more favourable hydraulic properties than BH4 and this is reflected in the lower upconing.

### 4.4. System Clogging and Decline in Produced Water

Over time, the produced water from BH2 drops from 30 L/s towards, and even below, 20 L/s (Figure 4). This is accompanied by a decline in drawdown in BH2 (i.e., rise in pumping water level), which indicates that the loss of yield is not solely due to a decline in specific capacity or efficiency of the abstraction borehole itself (which would be reflected in increased drawdown). Rather, it appears to be due to increased hydraulic resistance in the pipe—heat exchange—reinjection circuit.

**Figure 6:**
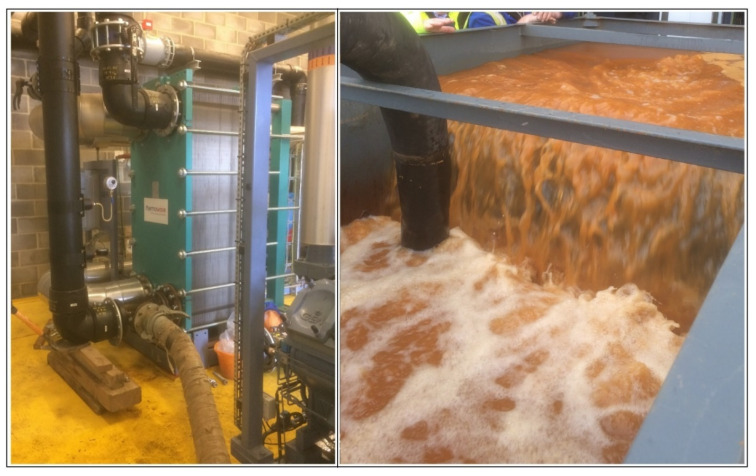
Recirculation of citric acid solution through the plate heat exchanger at Abbotsford Road, (**left**), to remove accumulated ochre (which can be seen in the recirculation tank, (**right**). Photos reproduced with permission of © David Banks, after [2,36].

On several occasions (21 September 2019, 6 December 2019, 14 December 2020, 17 May 2021), the plate heat exchanger (the most likely locus of clogging) has been coupled to a recirculation circuit via a tank and a solution of citric acid or oxalic acid has been circulated through the heat exchanger. The recirculated citric acid solution rapidly became loaded with orange dissolved and suspended iron (III) oxyhydroxide (Figure 6) that had accumulated in the heat exchanger (in the case of oxalic acid, the fluid tended towards a green colour, most likely ferric oxalate or ferrioxalate complex anions).

The flushing resulted in some recovery of system productivity—for example, on 6 December 2019 in Figure 4, when production improved from 23 to 30 L/s—but which immediately started to slowly decline. Repeated flushing of the plate heat exchanger on a twice-yearly basis appears to be necessary as an ongoing maintenance cost. Equipment lifting and CCTV inspection of abstraction borehole BH2 also revealed signs of ochre fouling on screen, rising main and pump in the form of a soft to firm sticky orange ferric oxyhydroxide deposit with some associated sand and sandstone grit. The borehole was thus cleaned using a proprietary oxalic acid-based product on 6 February 2020 (Figure 4) and the pump removed for cleaning. This appeared to result in a significant increase in yield, but this may also be ascribed to the permanent removal of a fine-mesh gauze filter installed within a plant room in-line dirt trap, which tended to become rapidly clogged and impede flow. The original capacity has never been permanently recovered with repeated flushing and rehabilitation, however, and at the time of writing (2021), the system is producing around 20–26 L/s of water. Recent inspection (2021) has also revealed a build-up of ochre within the header pipework at the reinjection wells, with some ochreous material in the reinjection wells; this has been described as soft, fine particulate ferric oxyhydroxide which was easily dislodged. The wells were cleaned by air-lifting and no permanent build-up of ochre or debris has been found on the walls or in the base of the reinjection wells.

### 4.5. Water Quality

Selected water quality data from Abbotsford Road are presented in Table 3. The water from the shallower boreholes BH1, BH2 and BH3, sampled between 2016 and 2018, was slightly brackish, with a typical electrical conductivity of 1300 µS/cm and a slightly alkaline pH. The waters are of Na-Ca-Cl-HCO_3_ type and are slightly reducing, as evidenced by the very low nitrate concentrations, but ammonium >1 mg/L. Concentrations of dissolved iron are variable but typically 250–350 µg/L, while dissolved manganese is around 150 µg/L. The sulphate/chloride mass ratio is considerably higher than the 0.1 typical of modern marine water, suggesting a lithological source of sulphate—most likely sulphide oxidation.

The deeper BH6, penetrating to the level of the High Main (E) seam, had an altogether different chemistry when sampled during initial test pumping in 2016, with saline water quality (electrical conductivity increasing from 3370 to 5520 µS/cm during constant rate testing in February 2016) reflecting elevated sodium and chloride concentrations. The pH is circumneutral. The chemistry of the water is Na-Cl type and appears to be more reducing than the shallow boreholes, with nitrate absent and ammonium concentrations of 2–3 mg/L. Iron was below detection limit in the water, but manganese was 1.2 to 1.3 mg/L. The sulphate/chloride ratio is around 0.03—much lower than the 0.1 typical of modern marine water. The low sulphate concentrations and absence of iron might suggest the onset of sulphate-reducing conditions. The subsequent operation of BH4, BH5 and BH6 as reinjection wells will have completely altered the local water chemistry in the High Main aquifer.

### 4.6. Tidal Fluctuations

**Figure 7:**
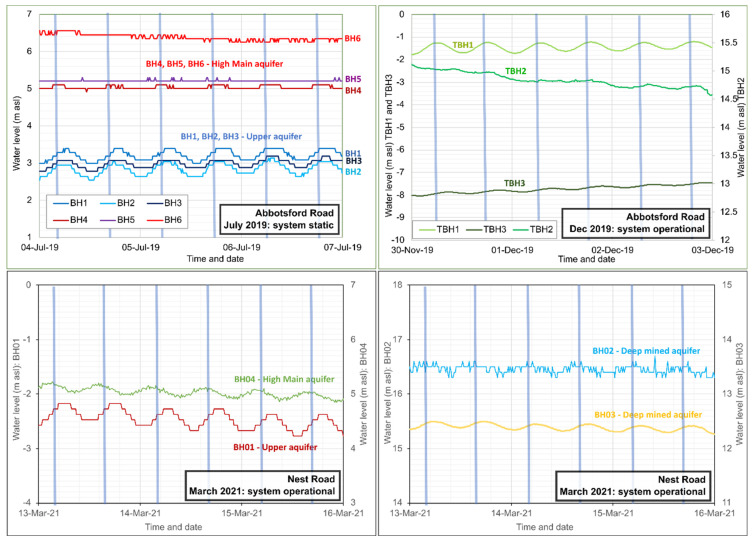
Tidal maxima recorded at the mouth of the Tyne Estuary at North Shields are shown as vertical blue bars. Hydrographs of specific periods have been selected based on (a) a good operational signal being received from all sensors, and (b) no interference from pumping switch-on/off. The selected periods are thus either static periods or periods of continual operation. The apparent “spikiness” or “quantisation” of some responses is related to the signal resolution logged by BMS.

The wells in the upper aquifer (TBH1, BH1, BH2 and BH3) exhibit clear tidal fluctuations (Figure 7), with an amplitude of up to 0.4 m, and the amplitude in TBH3 being somewhat less. In the deeper boreholes (BH4, BH5, BH6), the tidal variation is present, but at the limit of resolution of the data downloaded from the BMS sensors (0.1 m). The tidal variation lags somewhat after the estuarine tides measured at North Shields [14] in the shallower wells, but the lag appears slightly less in the deeper BH4. The tidal lag in the estuary itself between North Shields and Newcastle is reportedly very short [37], so it is assumed that any lag represents subsurface processes.

## 5. Nest Road

The Nest Road site (54.959° N 1.564° W) is a 2½ Ha plot around 700 m to the NW of the Abbotsford Road site. It is also occupied by a Lanchester Wines warehouse facility. It is located at an elevation of 24–25 m asl, around 300 m SW of the River Tyne estuary.

### 5.1. Boreholes

Four wells were drilled in 2016, as follows.

BH01 was drilled at 600–610 mm diameter to 49 m. A 500 mm OD uPVC casing was installed and grouted to 47.5 m depth. Drilling continued to 131 m at 450 mm nominal diameter. The hole passed through 42 m of superficial deposits, of which the upper portion was made ground and predominantly clayey material. The lower 16 m of the Quaternary sequence was described as “fine brown sand”. The grouted casing was insufficient to exclude this sandy material and it flowed into the hole. It was only possible to install an internal string of 315 mm OD/285 mm ID uPVC casing to 60 m depth, slotted from 40–60 m. Subsequently, the annulus outside the 315 mm casing was filled with backwashed 10 mm gravel to 41.5 m depth to prevent ingress of fine sand to above the top of the slotted casing. The borehole was thus not usable as a production well and was retained as an observation well with screen at shallow depth. The borehole did not encounter any coal workings.

BH02 was drilled at 600–610 mm diameter to 52 m, The borehole passed through 43 m of Quaternary superficial deposits (broadly clayey materials, with some sand and gravel, over fine brown sand, as in BH01). A 500 mm OD uPVC casing was installed and grouted to 47.5 m depth. The hole was continued at nominal 450 mm diameter to a reported 150 m depth. An internal (ungrouted) string of 315 mm OD uPVC casing was set to the base of the hole, with the lower section (40–150 m) slotted. Neither the High Main (E) coal nor any coal workings were recorded on the drilling log. After the current authors were engaged as consultants, the borehole was inspected in 2018, and was found to be 209 m deep. In May 2018, the borehole was deepened by diamond coring and reaming, to a total depth of 280.1 m. A drop-set string of uPVC casing (225 mm OD, 203 mm ID) was installed to 280.1 m depth, with a slotted section from 206 to 275 m. The intact Brass Thill (K) seam was encountered at 216 m, the intact Hutton (L) seam at 230 m and a zone of rubble representing goaf of the worked Harvey Beaumont (N) seam at 277 m.

BH03 was drilled at 600–610 mm diameter to 49 m. The borehole passed through 41 m of Quaternary superficial deposits, comprising predominantly Boulder Clay. A 500 mm OD uPVC casing was installed and grouted to 48 m depth. The hole was continued at nominal 450 mm diameter to 131 m depth. The High Main (E) seam was encountered at 114.5 m depth, with possible signs of workings. An internal (ungrouted) string of 315 mm OD uPVC casing was installed to 117.6 m depth, with the lower section (34–117.6 m) slotted. Due to poor yields (32 m cumulative drawdown at 5 L/s [38]), in summer 2018 the lower part of the borehole (including the High Main seam) was sealed by cement grouting and deepened at 270 mm using reverse-circulation water-flush techniques to 230 m. The extended hole intercepted intact coal seams as follows: Maudlin-Bensham (H) at 177 m, Six-Quarters (J) at 203 m, Brass Thill (K) at 212 m. A new internal string of 194 mm OD steel VAM casing was installed and grouted to 220 m depth, leaving the section 220–230 m open hole, corresponding to the horizon of the Hutton (L) seam at 224 m. Despite the seam being marked as worked on mine plans, the Hutton (L) seam was found to be apparently intact and significant yields were not achieved from the borehole.

BH04 was drilled at 600–610 mm diameter to 48.2 m. The borehole passed through 37 m of Quaternary superficial deposits, comprising predominantly Boulder Clay. A 500 mm OD uPVC casing was installed to 48.2 m and grouted to 45 m depth. The hole was continued at nominal 450 mm diameter to 131 m depth. An internal (ungrouted) string of 315 mm OD uPVC casing was installed to 130.4 m depth, with the lower section (61.8 to 130.4 m) slotted. The High Main (E) seam was encountered at 115 m depth, within a thick sandstone unit (High Main Post), extending from 93–121 m depth.

Thus, following drilling operations, only BH02 and BH04 were viable production wells. BH04 was hydraulically open from 48 to 131 m depth, although the High Main Post and coal were believed to be the predominant aquifer horizons. BH02 was hydraulically open from 48 to 280 m, although the only reported coal workings were at the Harvey Beaumont (N) level. We cannot, however, exclude that open coal workings were penetrated at the Maudlin-Bensham (H) or Six-Quarters (J) horizons in the unlogged portion of the borehole (150 to 209 m).

BH01 and BH03 were retained as monitoring points, with BH03 known to be hydraulically open only at the level of the Hutton (L) seam.

Rest water levels at the site were around +3 m asl (BH01) to +5 m asl (BH04) when the boreholes were first tested in 2016 [38].

### 5.2. Test Pumping

Initial test pumping in October 2016 was carried out [38] at rates of up to 30 L/s with abstraction from BH04 and reinjection to BH02. Drawdowns were minimal (c. 2 m) in BH04 and stabilised very rapidly. BH02 exhibited a significant “skin effect” (rapid step-up in water level on commencement of reinjection). Upconings reached 8 m after c. 2 days, but showed little sign of stabilising, and very slow recovery after cessation of pumping.

**Figure 8:**
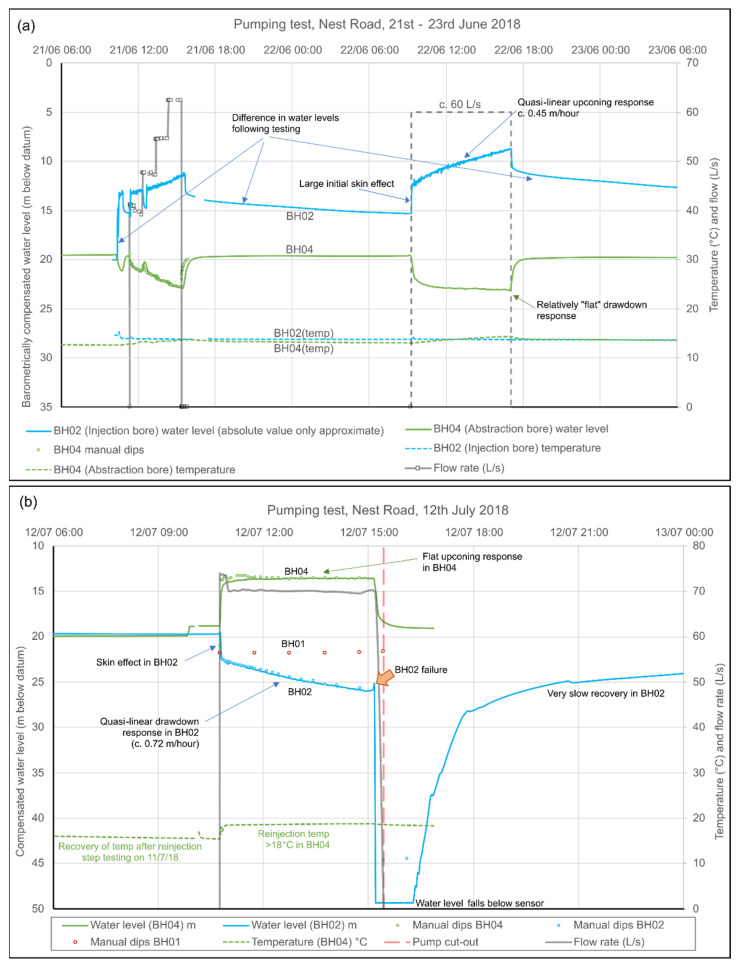
Hydrographs from short-term pumping tests of Nest Road BH02 and BH04 in (**a**) June 2018 (abstraction from BH04, reinjection to BH02) and (**b**) July 2018 (abstraction from BH02, reinjection to BH04). Water level barometrically corrected in m below surface. The temperature measured in BH02 is representative of the temperature at the sensor depth (c. 50 m) and not of in-situ deep mine water. Thus, in June 2018, the BH02 temperature reflects the reinjected water from BH04 during pumping. In July 2018, the BH04 temperature reflects the reinjected water from BH02 (18–19 °C).

Following the deepening of BH02 to attempt to improve its injectivity, BH02 and BH04 were hydraulically tested in abstraction and reinjection mode, in three series of tests, in June, July and October 2018 (Figure 8). In June 2018, BH04 was pumped with reinjection to BH02, with step testing followed by 8 hrs constant rate testing at 60 L/s. Despite the deepening of BH02, the same features were observed as previously: a significant skin effect (3 m at 60 L/s); an ability to accept significant reinjection, but without a tendency for upconing to stabilise; slow recovery after testing ceased. During reinjection, upconing in BH02 showed a quasi-linear trend increasing at an average of 10.8 m/day, with only a slow downward inflexion over time. This suggested the build-up of head in a fixed-volume underground reservoir, with little opportunity for excess head to disperse (at least, in the short term): i.e., high transmissivity (T), low storage (S), finite reservoir. Drawdown in BH04 was low (c. 3.5 m) and appeared to “flatten” very rapidly (specific capacity 1480 m^2^/d). Abstracted water temperatures were 13–14 °C during testing.

Testing in July 2018 comprised step testing, followed by 4½ h constant rate testing at c. 70 L/s, with pumping from BH02 and reinjection to BH04. The water temperature appeared to be 18.5 °C, suggesting a depth of origin near the base of borehole BH02. The results were essentially the inverse of the June 2018 testing. BH02 exhibited a significant skin effect (immediate drawdown of almost 3 m) followed by a quasi-linear increase in drawdown (at around 17 m/day), which reached 6.3 m with no sign of stabilising. BH04 exhibited an upconing of 5.2 m, which appeared to flatten relatively rapidly (specific capacity 1160 m^2^/d). After c. 4.5 h pumping, however, a catastrophic ingress of fine sand/silt occurred to BH02 (presumably Quaternary superficial sand from behind an inadequately grouted casing) and the test was terminated. This necessitated airlifting of the borehole to remove the sand. Subsequently, BH02 has not been pumped, but merely been used as a reinjection borehole: no repeat of the sand ingress has been observed.

Testing in October 2018 comprised step testing, followed by 4 days constant rate testing at 40 L/s, with pumping from BH04 and reinjection to BH02 (abstracted water temperature 13.0 to 13.2 °C). BH02 still exhibited a large skin effect (c. 10 m, i.e., larger than in June 2018), and a very slow post-test recovery, and also a quasi linear upconing trend. However, compared to June 2018, the rate of upconing had reduced significantly to between 0.6 and 4.4 m/day. Overall, heads rose in BH02 to within 4 m of the surface. BH03 exhibited a lower absolute upconing than BH02, but also with a linear rate of rise (1.77 m/d). As before, drawdown in BH04 was low (2.2 m) and appeared to “flatten” very rapidly.

Calculated specific capacities at BH04 from the various test episodes range from 1160 to 2160 m^2^/d, suggesting a much higher transmissivity than observed at Abbotsford Road.

### 5.3. Operational Monitoring

**Figure 9:**
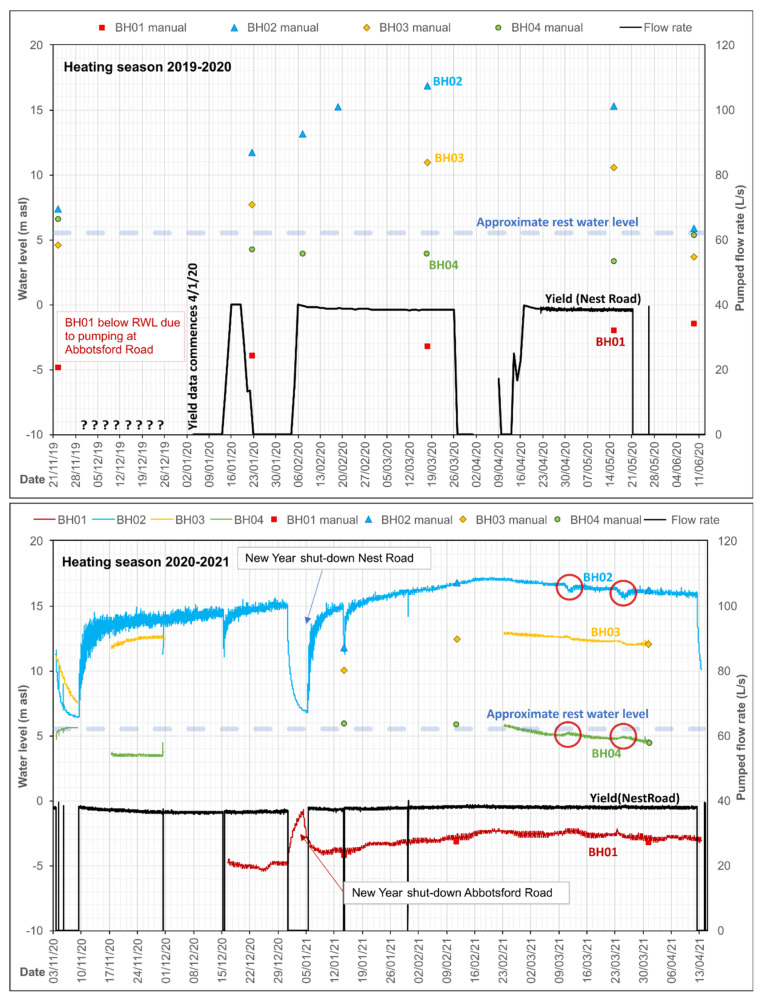
Hydrographs from operation of the Nest Road scheme during the periods November 2019 to June 2020 (manual data, **top**) and November 2020 to April 2021 (mostly automatically logged data, **bottom**). The system was typically non-operational during summer. Replacement of sensors means that only limited logged data are available for BH03 and BH04. Note that BH01 records a large drawdown even when Nest Road is non-operational (November 2019), in response to pumping at Abbotsford Road. Red circles show responses to test pumping at Gateshead Baltic minewater thermal project.

Despite the limited injection capacity of BH02 during 2018 testing, recent (2020–2021) operational long-term pumping of BH04 and reinjection to BH02 at rates of 37–40 L/s have demonstrated that BH02 is quite capable of accepting these quantities of water for a prolonged period, without the water level rising beyond 6 m of the well top. The behaviour of the borehole and/or aquifer thus seems to have improved between October 2018 and 2020. Selected operational groundwater monitoring data are presented in Figure 9.

BH03 exhibits a rise in groundwater head in response to operation, and effectively represents a subdued version of the injection response in BH02 with little or no “skin effect” (upconing due to well inefficiency). BH03 is believed to be in close proximity to mine workings in the Hutton (L) seam, which are in turn connected by drifts and shafts to other worked seams of Felling Colliery, including the Harvey-Beaumont (N), intercepted by BH02.

BH04 continues to exhibit minimal drawdown (<3 m at 40 L/s) in response to pumping. The reducing, H_2_S-rich nature of the water has proven highly aggressive towards conventional downhole pressure/temperature loggers (hence the limited logged data in Figure 9) and has necessitated replacement with corrosion-resistant titanium loggers.

BH01 exhibits a groundwater head well below “rest” level, even when Nest Road is non-operational. In fact, BH01 does not respond to operations at Nest Road, but rather exhibits a large drawdown response (up to c. 8 m, see Figure 5) to abstraction at Abbotsford Road. Its response correlates very well with TBH1 at Abbotsford Road. In the lower part of Figure 9, for example, it can be seen that, during the cessation of pumping at New Year at Nest Road, BH01 starts to respond to the earlier start-up of Abbotsford Road in the morning of 4 January 2021, whereas Nest Road did not start operating until noon on 5 January 2021. Thus, water levels in BH01 are drawn down to below the dynamic water level of any of the operational boreholes at Nest Road. BH01 thus appears to respond hydraulically to heads in the “Upper Aquifer”—the sandstone horizons above the High Main Post, abstracted by the wells at Abbotsford Road. There appears to be no hydraulic connection with the High Main aquifer or deeper mined aquifers. Drawdown at Nest Road BH01 is thus included in Figure 5.

To date, no remedial work has been required at Nest Road, due to clogging of wells, pipes or heat exchangers.

### 5.4. Water Quality

#### 5.4.1. Borehole BH02

Groundwater abstracted from BH02 in July 2018 has not been analysed at a laboratory, but field determinations were recorded during the pumping test of July 2018: the electrical conductivity was up to 27,780 µS/cm (close to the conductivity of seawater [39]), with a pH of 6.3 to 6.5.

#### 5.4.2. Borehole BH04

The water from BH04 has an H_2_S odour. Its chemistry has changed through time, with salinity and sodium-chloride concentrations increasing (Table 4).

When initially test pumped in 2016, the pH was sub-neutral (c. 6.5), with around 500 mg/L chloride, 350 mg/L sodium and 260 mg/L sulphate. The water was of Na-(Ca)-HCO_3_-Cl type.

By the tests of September–October 2018, chloride had jumped up to 1600 mg/L and sodium to 690–970 mg/L, although there had been injection of saline water from BH02 to BH04 a few months earlier in the summer, which might partially explain the residual salinity. Sulphate had declined a little to <200 mg/L. The water had thus become a more brackish Na-Cl type.

Samples taken in April 2019 revealed that chloride was still 1200–1400 mg/L and sodium around 900 mg/L. More interestingly, sulphate had disappeared from the water, suggesting the onset of strongly sulphate-reducing conditions. This is corroborated by the rather high barium concentrations of up to 1.9 mg/L. pH was now around 7.5. Lithium and strontium were also elevated at c. 1.5 and 4.7 mg/L respectively.

Iron has consistently been relatively low in the water from BH04 at <0.5 mg/L with manganese typically a little higher. Finally, it is worth noting that the temperature of water from BH04 in April 2019 was 13.8 °C, but only 13.3 °C when first tested in 2016.

Taken together, this suggests that BH04 is progressively drawing on a greater component of deeper (possibly warmer) more saline, more reducing water. The increased salinity is unlikely to be due to saline intrusion from the Tyne Estuary, as the Li/Cl and Sr/Cl mass ratios in sea water are 5 to 9 × 10^−6^ and 7 × 10^−4^, respectively, while in BH04, the ratios are between 1 × 10^−3^ and 3–4 × 10^−3^ [40,41,42].

### 5.5. Tidal Fluctuations

All wells at Nest Road exhibit tidal fluctuations (Figure 7): the deepest boreholes BH02 and BH03 exhibit a range of up to 0.2 m, BH04 exhibits an amplitude of just over 0.2 m and BH01 exceeding 0.4 m. The tidal variations in BH02 and BH04 approximately coincide with the estuarine tides measured at North Shields [14] while BH03 and BH01 lag behind—by up to 3.5 h in the case of BH01.

## 6. Interference between Abbotsford Road and Nest Road

During the December 2017 testing [35] at Abbotsford Road, the water levels in the newly drilled BH04 at Nest Road (into the High Main (E) seam) appeared to show a response to reinjection at Abbotsford Road, with water levels increasing by up to c. 0.5 m, coinciding with Abbotsford system switch-on.

**Figure 10:**
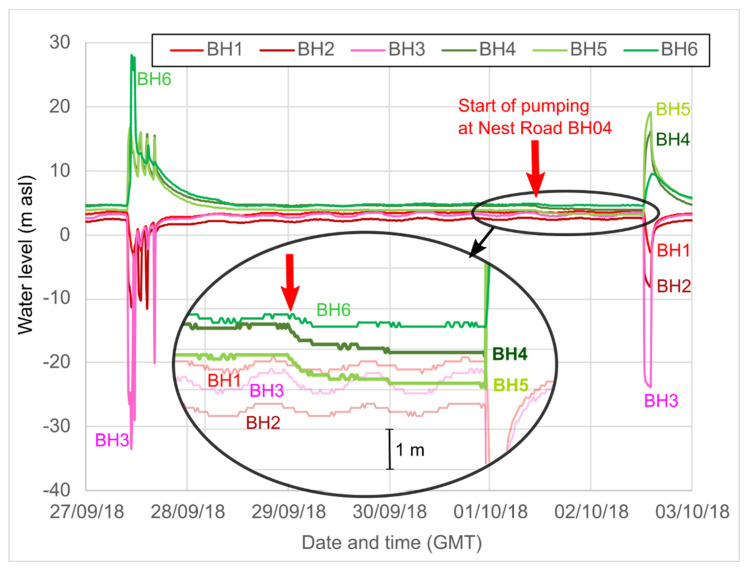
Water levels (m asl) logged by the building management system (BMS) at Abbotsford Road, October 2018. The elliptical inset shows an enlargement of the response to the start of the pumping test (shown by vertical red arrow) at Nest Road BH04 (c. 735 m away from the Abbotsford reinjection wells) with reinjection to BH02. Note also the large tidal fluctuations in Abbotsford BH1 to BH3.

There was also a clear response at Abbotsford Road to the pumping test carried out at Nest Road BH04, commencing at 11:34 a.m. on 1 October 2018 (Figure 10). On 1 October, Abbotsford Road was not operating and a Theis-like response was observed, in the three injection boreholes (BH4 to BH6) at Abbotsford Road, but not in the shallower abstraction boreholes (BH1 to BH3). The greatest responses were observed in BH4 and BH5 (c. 0.8 m drawdown), although the response became obscured by operation of Abbotsford Road on 2 October.

In addition, there is evidence of hydraulic interaction between Nest Road and the new Gateshead Baltic district minewater heating scheme being developed near Shearlegs Pit, some 1.9 km WNW of Nest Road ([11], Figure 3). Exploratory boreholes connected with this scheme were being drilled, test pumped and monitored during early 2021. On 12 April 2021, a minor equipment failure caused a pump shut-down at Nest Road. The Coal Authority (*Mr J Gordon, pers. comm.*) immediately observed (i) a > 3 m decline in head in a Hutton (L) seam monitoring borehole at Gateshead Baltic and (ii) a c. 0.5 m rise in water level in a High Main (E) seam monitoring borehole. Such a strong, immediate response over such a large distance implies (i) a very high hydraulic diffusivity (ratio of transmissivity to storage) in the workings of both the High Main and the deeper seams, (ii) a highly confined (low storage) response in the deeper workings associated with the Hutton (L) seam. Moreover, test pumping of the Hutton (L) seam at Gateshead Baltic, with reinjection to the High Main (E), on 10 and 25 March 2021, produced responses in Nest Road BH02 and BH04 (albeit of a smaller magnitude, reflecting the lower hydraulic stresses applied during the pumping test at Gateshead Baltic). These are circled in red in Figure 9. This interference is currently being examined and will doubtless be reported more fully in due course.

No evidence of interference has been observed between the reinjection well (BH02) at Nest Road and any well at Abbotsford Road, nor has any mutual hydraulic interference been observed between the abstraction well (BH2) at Abbotsford Road and the abstraction well at Nest Road (BH04). Abstraction from Abbotsford Road BH2 causes a significant drawdown, and immediate response in Nest Road BH01.

Although Abbotsford Road reinjects cooled water to the horizon of the High Main seam via BH4 and BH5, and despite a modest hydraulic response being observed in the High Main abstraction borehole at Nest Road (BH04), no convincing evidence of thermal breakthrough has yet been observed in the water abstracted at Nest Road BH04.

## 7. Conceptual Hydrogeological Model

It is the opinion of the authors that too little hydrogeological consideration was given to the original design of the schemes at Abbotsford and Nest Road. The initial expectation of yield from the Coal Measures aquifer at BH1, BH2 and BH3 was extremely optimistic, although the aquifer did prove to be remarkably productive in yielding >30 L/s from individual boreholes in what is widely regarded as a “minor” aquifer [43]. However, the boreholes were too closely spaced to avoid significant mutual hydraulic interference; the abstraction of 20–30 L/s from the best borehole (BH2) caused significant drawdown in the other two boreholes and eventually, operation of more than one borehole (BH2) at a time produced little in additional yield. BH1, BH2 and BH3 did not penetrate the High Main Post or High Main (E) seam; the aquifer horizons accessed by these boreholes are informally termed the Upper Aquifer System (UAS). The transmissivity of the UAS is likely to be dominated by sandstone horizons (possibly including the Seventy Fathom Post, although it is difficult to identify this stratigraphic unit unambiguously in the drilling logs). Test pumping has demonstrated that monitoring boreholes TBH1 and TBH3 also respond to abstraction from BH2 and are thus also assigned to the UAS. Test pumping suggests that the UAS has a transmissivity of around 100 m^2^/d (Figure 5). It is, however, both inhomogeneous (evidenced by the differing yields of BH1, BH2 and BH3) and anisotropic (evidenced by the similar long-term drawdowns in Nest Road BH01 and Abbotsford Road TBH1, despite very different distances from BH2, Figure 5). Strong inhomogeneity and anisotropy are well recognised in the Coal Measures aquifer system in Great Britain [44].

The deeper injection boreholes at Abbotsford Road penetrated the High Main Post sandstone unit and the High Main (E) seam, which is recorded as being historically worked via pillar and room techniques from Felling Colliery, although workings were only identified in one of the three boreholes (BH4). Two of the injection wells (BH4 and BH5) were capable of accepting significant quantities of water (>15 L/s each), with the injectivity of BH6 being significantly lower. We interpret BH4 and BH5 as accessing a different, deeper aquifer unit to BH1, 2 and 3, which we refer to as the High Main Aquifer System (HMAS). The HMAS encompasses the workings of the High Main (E) coal seam and the overlying High Main Post, which may be fractured due to partial collapse of coal pillars in the High Main (E) seam or, in some cases, total collapse caused by pillar robbing and total extraction working. BH6 does respond hydraulically to injection in BH4 and BH5, as does the deep monitoring borehole TBH2: these are also assigned to the HMAS. These boreholes are, however, also hydraulically open through the overlying UAS: their hydraulic response is, however, dominated by upconing and injection to the HMAS. We thus cannot wholly exclude the possibility that this response is counterbalanced by a minor component of drawdown due to abstraction from the UAS. The fact that the response in TBH1 is clearly an abstraction response to pumping of BH2, despite being so close to the injection boreholes, demonstrates (a) that the injection boreholes’ interaction with the UAS is very low and (b) that there is very poor vertical hydraulic connectivity between the UAS and the HMAS.

At Nest Road, BH04 penetrates the High Main Post and the High Main (E) seam. There is documented hydraulic interference between BH04 and Abbotsford Road BH4 and BH5, but not with the abstraction boreholes (BH1, BH2 and BH3). We thus conclude that Nest Road BH04 is abstracting primarily from the HMAS (either workings in the High Main (E) seam or fractured High Main Post). The hydraulic response of Nest Road BH04 is very different to that of Abbotsford Road BH4 and BH5, however: the former yields extremely well (70 L/s with minimal drawdown), with a very flat drawdown response, while Abbotsford Road BH4 and BH5 accept up to c. 20 L/s with a significant upconing and a more conventional Theisian response. Although the boreholes access the same aquifer unit, the limited mutual hydraulic interaction (<0.8 m drawdown at Abbotsford Road BH5 due to pumping at Nest Road BH04) and the different hydraulic responses suggest some form of hydraulic discontinuity between the sites within the HMAS. The relatively flat response and low drawdown at Nest Road BH04 suggest a high transmissivity (>1000 m^2^/d on the basis of specific capacity) and a possible presence of a positive constant head or “leaky” boundary condition: this could be the Tyne Estuary (via a significant thickness of intervening alluvial, glaciofluvial and Coal Measures deposits), leakage from over-/underlying horizons, or simply the proximity of a large network of highly transmissive, widely interconnected mine voids (drawdown effectively being limited by the proximity of a large underground “lake” of flooded mines). It should be stressed that the presence of a constant head boundary condition does not necessarily imply induced recharge (e.g., from the Tyne); drawdown can also be suppressed by interception of discharge to such a boundary.

Although chilled water is being reinjected to the HMAS at Abbotsford Road, and water is being abstracted at Nest Road from BH04 some 735 m away, there is thus far no clear evidence of chilled water appearing at the Nest Road abstraction. It is arguably too early to conclude that there is a lack of thermal interference, however, and monitoring for signs of thermal breakthrough will continue.

BH02 intercepted mine workings at the Harvey-Beaumont (N) horizon when it was deepened in 2018. Although workings were not encountered in the Hutton (L), it is known that this seam was intensively worked in the vicinity. BH02 may also have intercepted workings in the Maudlin-Bensham (H) or Six-Quarter (J) seams prior to deepening. The borehole is capable of accepting injection flows of at least 60–70 L/s. However, test pumping suggested that injectivity was ultimately limited by head build-up over time, without significant dispersion of excess heads. Despite encountering deep open workings, the injectivity (in terms of head build-up) did not clearly improve upon deepening. These observations imply that (a) the injectivity at BH02 is limited by storage, rather than transmissivity and (b) that the reservoir intercepted by BH02 is limited in extent: exactly what one might expect from a deep mine working. If one takes the rate of drawdown during the July 2018 test of c. 0.72 m per hour for a pumping rate of 70 L/s = 252 m^3^/h, then for every m decline in head (10,160 Pa decline in pressure assuming a seawater density), around 350 m^3^ water are being released, equating to a figure of 0.0344 m^3^ Pa^−1^. Given that the compressibility of water is 5 × 10^−10^ Pa^−1^ [45], this suggests a reservoir volume of around 69 million m^3^, if the quasi-linear nature of the drawdown in Figure 8 is solely due to depletion of a finite reservoir of water. Assuming a cumulative thickness of worked seams (Maudlin (H) to Harvey (N)) of 5 m, this equates to an average reservoir area of 14 million m^2^ or 14 km^2^, which is not wholly unreasonable in the context of Figure 3. If one accepts that part of the compressibility is also due to rock matrix compression or some movement of a free water surface, the estimated area reduces further.

Mine plans suggest that worked seams from the Maudlin-Bensham (H) down to the Harvey-Beaumont (N) are rather well interconnected by drifts and shafts, but that these deeper seams are not especially well-connected with the shallower High Main (E) seam. We thus postulate that BH02 accesses a Deep Mined Aquifer System (DMAS), characterised by high transmissivity, but low storage and limited areal extent, which is hydraulically separate from the UAS and HMAS. This is supported by the documented hydraulic interference with the Gateshead Baltic scheme, implying a large hydraulic diffusivity (high T, low S).

Nest Road BH03, as deepened in 2018, was designed to intercept the Hutton (L) seam. While it did not encounter worked voids, it clearly responds to injection at BH02 and seems to enjoy some connectivity to the (presumed nearby) mined Hutton seam. It is thus assumed to monitor heads in the DMAS.

Nest Road BH01 responds to pumping at Abbotsford Road BH2 and is thus assigned to the UAS. Indeed, the magnitude of the drawdown response is so great that it is comparable to that observed in Abbotsford TBH1.

Tidal variations of groundwater head are greatest in the Upper Aquifer and lowest in BH02 (Deep Mined Aquifer). The UAS boreholes also exhibit the greatest temporal lags from the estuarine tide cycle, while the lags are lower in the deeper aquifers. Tidal cycles in groundwater head can be due to (i) earth tides, (ii) direct hydraulic interaction with a tidal water body (the Tyne Estuary) or (iii) pressure loading from a tidal water body. In the case of Abbotsford Road and Nest Road, we believe earth tides to be unlikely as a cause, as the deepest aquifer units exhibit a response correlating rather well with the estuarine tides, whereas real estuarine tides would lag behind “true” earth tides by a factor determined by geography. We also believe that the vertical distance of the mined seams below the bed of the Tyne, coupled with the documented extremely low vertical hydraulic connectivity of the Coal Measures sequence, makes direct hydraulic connection with Tyne an unlikely scenario for the HMAS and DMAS. Tidal pressure loading from the Tyne and subsequent compression of aquifers and mine workings would appear to be the most likely explanation for tidal cycles in groundwater head in the HMAS and DMAS (high diffusivity due to high T and low S causes immediate tidal response, but decreasing amplitude with depth). The tidal response in the UAS may also be due to tidal loading, but could also be due to a component of direct hydraulic connection. The larger, but delayed, response reflects the less confined nature of the aquifer and its likely lower transmissivity (not comprising open mined voids), resulting in a lower hydraulic diffusivity.

The documented hydraulic interference in the HMAS between Nest Road and Gateshead Baltic is arguably expected, given the extensive occurrence of pillar and room workings (likely open voids) beneath large areas of Newcastle and Gateshead (Figure 1). More surprising is the large hydraulic interference seen in the DMAS, given that the workings of Felling Colliery and Tyne Main/Shearlegs Collieries at the level of the most extensively worked deep seams (e.g., Hutton (L)—Figure 3) are largely separated by a barrier of unworked coal, breached only by individual roadways. However, the response does demonstrate that a hydraulic signal can be efficiently transported across a large distance in a highly confined mined aquifer, provided that some hydraulic connections remain open. It should be emphasized that such a hydraulic (pressure) response does not necessarily imply any large-scale exchange of water or heat between the two areas of mine workings.

The water chemistry data suggest that groundwater salinity increases rapidly with depth in the Coal Measures aquifer and becomes strongly reducing. Water from the UAS (Table 3) contains around 100 mg/L sulphate, 200–300 mg/L chloride, negligible nitrate, around 1 mg/L ammonium and modest quantities of dissolved iron (a few hundred µg/L) and manganese (100–200 µg/L). This suggests that the waters are mildly reducing (iron/manganese/nitrate reducing) but not sulphate reducing. They are also mildly affected by salinity, either from intrusion of estuarine water or upwelling of deeper Coal Measures groundwater.

At Abbotsford Road, the waters of the HMAS, on initial testing, were significantly more saline (1000 mg/L or more chloride), but poorer in sulphate (40–60 mg/L), with negligible iron and with significantly higher concentrations of manganese (>1000 µg/L) and ammonium (2–3 mg/L) than the UAS, suggesting a redox conditioning becoming sulphate-reducing. The HMAS at Nest Road (as represented by BH04, Table 4) is similar in chemistry, albeit with slightly more iron and less manganese and ammonium. With time, the water quality at Nest Road BH04 has become increasingly saline and increasingly reducing. The elevated Sr/Cl and Li/Cl suggest that the salinity is derived from deeper formation waters rather than intruding estuarine water. Initial sulphate concentrations of c. 200 mg/L have disappeared over time, with the onset of sulphate-reducing conditions, detectable H_2_S (which has caused instrument corrosion) and elevated barium (which only becomes soluble in the absence of sulphate).

Regrettably, the chemistry of BH02 was not thoroughly documented before being commissioned as an injection borehole, but field data shortly after deepening suggest a salinity approaching seawater.

As regards the sites’ behaviour in relation to the area’s regional hydrogeology, both the Coal Authority and Environment Agency operate a number of observation boreholes (OBH) in the vicinity of the site, particularly on the north bank of the River Tyne in the Walker area (Figure 1 and Figure 3). Despite the potential for hydraulic interaction via mined High Main (E) workings beneath the River Tyne (Figure 3), hydraulic impacts from the operation of the Abbotsford/Nest Road schemes have not hitherto been detected in these regional observation wells, although it is acknowledged that not all of the wells are monitored at a frequency suitable to detect such impacts. The regional observation wells all exhibit water levels similar to those at Nest/Abbotsford Road (+3–5 m asl). It is believed that the Nest and Abbotsford Road sites lie within the so-called Walker Block [46]—a mine water catchment likely draining via diffuse seepage, towards the Tyne Estuary. Springwell Quarry OBH (54.9214° N 1.5578° W), to the Hutton (L) seam, some 4 km to the south of Felling, exhibits a water level of c. +7 m asl, suggesting the existence of a shallow watershed between the Walker Block and flooded mine workings to the south of Springwell Quarry, which drain southwards towards the regional discharge points of Chatershaugh (gravity discharge at 54.8759° N 1.5199° W, +6 m asl [47]) and Kibblesworth (pumped discharge at 54.9002° N 1.6217° W [48,49,50]).

## 8. Conclusions

In summary, the data collected at Abbotsford Road and Nest Road allow us to hydrogeologically conceptualise the area as a stack of (at least) three aquifer systems, which are largely hydraulically independent:

- An Upper Aquifer System (UAS), comprising the Seventy Fathom Post and associated sandstones (and possibly fractured siltstones and mudstones), with a transmissivity of around 100 m^2^/d. There is evidence of lateral anisotropy and inhomogeneity. The aquifer contains slightly brackish, sulphate-rich, mildly reducing groundwater, with a significant iron content.
- A High Main Aquifer System (HMAS), comprising the (possibly fractured) High Main Post and workings of the High Main (E) coal seam. The aquifer contains brackish, reducing water, on the threshold of sulphate-reduction. At Nest Road, prolonged abstraction has resulted in draw-up of deeper saline water, and pushed the water into the sulphate-reduction redox field. The hydraulic response at Nest Road is high-transmissivity and indicative of a nearby constant head or leaky aquifer boundary: this could be the River Tyne (via intervening sediments), leakage from over- or underlying aquifer units, or proximity to a regionally extensive, high transmissivity network of mine voids. The HMAS hydraulic response at Abbotsford Road appears lower transmissivity and more conventional (Theisian). The High Main (E) seam workings are regionally extensive and connect NW to the areas of Tyne Main and Shearlegs collieries and below the Tyne.
- A Deep Mined Aquifer System (DMAS) comprising the workings of the Harvey-Beaumont (N) and Hutton (L) seams, and most likely the overlying Brass Thill (K), Six-Quarters (J) and Maudlin-Bensham (H) seams. The water is saline and likely to be highly reducing. The hydraulic response is highly confined and was (during testing) indicative of an aquifer of limited volume and limited capacity to disperse excess head. During operation, BH02 at Nest Road seems able to disperse at least 40 L/s water sustainably, indicating that either (i) initial testing had underestimated the ability of the aquifer to disperse excess head, (ii) under prolonged operation, water “found” pathways by which to disperse excess head, or (iii) the nature of aquifer has changed between testing and prolonged operation (e.g., changes in mined passages or drilling at other sites having penetrated the aquifer allowing dispersal of head).

The following observations from the Nest Road and Abbotsford Road sites should assist future developers in designing and constructing MGES schemes [2]:

1. It is essential that experienced hydrogeologists are involved in the design of mine water geothermal schemes, to avoid issues of well interference and to avoid unrealistic expectations of yield.
2. It is essential that experienced groundwater engineers are involved in the design of mine water geothermal schemes, to advise on and supervise appropriate well design and construction.
3. With older mine plans, and especially at depth, it is unreasonable to expect to be able to reliably target a mine void with a single borehole. One might expect to drill 2-3 boreholes into pillar and room mine workings in order to obtain a “good” borehole (which corresponds with commonly cited ratios of room/pillar). The likely verticality of boreholes should also be considered in this context and appropriate drilling techniques selected [2].
4. It is highly challenging to predict the behaviour of mined aquifer systems prior to drilling and testing—in this example, it would have been almost impossible to predict the “flat”, low drawdown response of Nest Road BH04, or the “sealed reservoir” response of BH02 prior to drilling, or to realistically predict the degree of hydraulic interaction between the systems. A significant degree of uncertainty (and thus risk) must be accepted when designing and planning mine water geothermal schemes.
5. Wells with large exposed open sections should be avoided (at Abbotsford Road, this was challenging, due to the shallow nature of the Upper Aquifer, and modest transmissivity resulting in large drawdowns). Cascading of water within wells is likely to promote iron oxidation and subsequent precipitation.
6. The importance of data collection (temperature, basic chemistry, electrical conductivity, head) is emphasised, at an appropriate frequency, precision and resolution. A program of regular manual dipping and sampling is recommended to calibrate automated measurements. Data should be regularly collated, quality controlled and archived. Developers of conventional building management systems (BMS) may require some education in the significance of downhole data and regular calibration routines.
7. Mine water chemistry can be highly aggressive and suitable well construction, pipeline, heat exchanger and sensor materials should be selected.
8. Progressive scaling or clogging with iron oxyhydroxides is a very common (though not inevitable [2]) occurrence in mine water geothermal systems. This can occur in abstraction or reinjection wells, heat exchangers or pipelines. The regular documentation of well specific capacity (e.g., on Q/s diagrams [51,52,53]), and monitoring of pressure losses in pipelines, heat exchangers and reinjection mains is essential.
9. Operators should not expect mine water geothermal systems to involve minimal maintenance. Indeed, maintenance requirements can be high and may involve regular emptying or backwashing of filters, chemical flushing of heat exchangers and pipelines, replacement and calibration of sensors and even rehabilitation of boreholes, when specific capacity or injectivity falls below a certain trigger value. This ongoing economic burden may mean that only relatively large minewater geothermal schemes, with an assured income and budget, will be economically sustainable.

## Figures and Tables

**Table 1:** Summary of the Abbotsford Road and Nest Road mine water geothermal energy schemes. UAS = Upper Aquifer System, HMAS = High Main Aquifer System; DMAS = Deep Mined Aquifer System.

Scheme	Abbotsford Road	Nest Road
Grid reference	54.955° N 1.556° W	54.959° N 1.564° W
Heating or cooling?	Heating only	Heating only
Installed heat pump capacity	2.4 MW	1.2 MW
No./depth of functioning production wells	1 to 110 m	1 to 131 m
No./depth of functioning reinjection wells	2 to 155 m	1 to 280 m
Production horizon	UAS	HMAS
Reinjection horizon	HMAS	DMAS
Typical current operational rate	20–30 L/s	40 L/s
Abstracted water temperature	11–13 °C	13–14 °C
Maximum licensed yield	49 L/s	67 L/s

**Table 2:** The main features of strata encountered in the Venture Pit shaft (and projected down to Harvey-Beaumont ‘N’ seam), within the Nest Road site, using data from BGS record NZ26SE83 and [22]. Quaternary superficial material was 40.2 m thick in the shaft. The High Main Post sandstone extended from 98.8 to 118.4 m bgl (m asl = m above sea level, m bgl = m below ground level).

Horizon	Alternative Name		m asl	m bgl	Extraction Method below Nest Road
Surface			22.9	0.0	
**Quaternary Drift sequence**					
Soil and clay	to		−12.2	35.1	
Sand	to		−17.4	40.2	
**Coal Seams**					
Threequarters	Ryhope Five-Quarter	C	−30.5	53.3	Unworked in vicinity of shaft
High Main (E)		E	−97.2	120.1	Pillar and stall
Metal	Top Main	F1	−107.8	130.7	Unworked in vicinity of shaft
Stone/Main	Five Quarter	F2	−114.6	137.5	Unworked in vicinity of shaft
Yard		G	−130.9	153.8	Unworked in vicinity of shaft
Bensham	Maudlin	H	−156.7	179.5	Pillar and stall/total extraction
Six-Quarter	Durham Low Main	J	−182.3	205.1	Pillar and stall
Five Quarter	Brass Thill or Northumberland Low Main	K	−191.2	214.1	Unworked in vicinity of shaft
Hutton	Low Main	L	−203.8	226.7	Pillar and stall
Shaft depth (1801)			−206.8	229.7	Unworked in vicinity of shaft
**Projected Seams below Shaft Base**					
Harvey	Beaumont	N	−248.6	271.5	Total extraction (pillar and stall)

**Table 3:** Selected water chemistry data from pumping of the Abbotsford Road boreholes (CRT = sampled during constant rate testing). See Figure 2 for borehole locations.

Borehole		BH1	BH1	BH2	BH2	BH3	BH3	BH6	BH6
		CRT						CRT	CRT
Date (dd/mm/yy)		18/2/16	27/7/17	03/6/16	27/7/17	03/6/16	09/1/18	24/2/16	25/2/16
**Physico-chemical parameters**									
Conductivity @ 20 °C	µS/cm	1260	1410	1330	1400	1770	1240	3370	5520
pH		7.68	7.73	7.94	7.68	7.74	7.60	7.69	7.59
Anions									
Nitrate as NO_3_^−^	mg/L	0.42	<0.3	<0.3	<0.3	<0.3	<0.3	<0.3	<0.3
Sulphate as SO_4_^=^	mg/L	84.7	134	101	139	70	135	40.3	63.5
Chloride	mg/L	205	234	195	235	380	191	962	1860
Alkalinity, Total	meq/L	6.79	7.19	7.09	6.39	6.39	6.99	6.99	6.79
**Cations (dissolved filtered)**									
Calcium	mg/L	86.5	110	88.9	107	101	102	196	298
Sodium	mg/L	115	147	134	143	189	125	507	944
Magnesium	mg/L	32.3	45.2	35.3	44.7	41	41.3	58.1	102
Potassium	mg/L	17	16.9	15.8	16.6	15.9	15.7	29.7	32.8
Ammoniacal-N as NH_3_	mg/L	1.17	0.98	1.24	1.18	1.17	0.93	3.26	2.33
Iron	mg/L	<0.02	0.28	-	0.35	-	0.25	<0.02	<0.02
Manganese	μg/L	114	159	-	161	-	135	1230	1290
**Cations (total unfiltered)**									
Iron	mg/L	-	6.7	-	0.19	-	0.21	-	-
Manganese	μg/L	-	184	-	175	-	145	-	-

**Table 4:** Selected water chemistry data from pumping of (first column) Nest Road BH03 prior to deepening and BH04 (End Step = end of step of step testing, CRT = sampled during constant rate testing, - = not analysed). Dissolved oxygen in BH04 has been consistently <0.3 mg/L and suspended solids <3 mg/L. Early data derived from [38]. Borehole locations are shown in Figure 2.

Borehole		BH03	BH04	BH04	BH04	BH04	BH04	BH04	BH04
		End step	End step	CRT	CRT				
Date (dd/mm/yy)		28/9/16	20/9/16	23/9/16	26/9/16	25/9/18	05/10/18	16/4/19	16/4/19
**Physico-chemical parameters (measured in field)**									
Conductivity	µS/cm	3220	2380	2430	2500	4500	4590	5407	5466
Temperature	°C	13.3	13.3	13.3	13.4	14.2	13.2	13.8	13.8
pH		6.6	6.4	6.5	6.5	6.1	6.8	7.5	7.5
**Anions**									
Nitrate as NO_3_^−^	mg/L	<0.3	<0.3	<0.3	<0.3	0.58	0.60	2.9 ?	2.9 ?
Sulphate as SO_4_^=^	mg/L	295	268	244	265	119	190	<0.19	<0.19
Chloride	mg/L	735	435	486	505	1600	-	1200	1400
Alkalinity, Total	meq/L	11.0	11.4	11.4	11.4	-	-	11.0	10.8
**Cations (dissolved)**									
Calcium	mg/L	150	146	134	152	190	174	150	150
Sodium	mg/L	552	330	343	376	970	690	940	960
Magnesium	mg/L	48.5	62.7	58.9	65.2	84	84	66	67
Potassium	mg/L	20.6	22.7	21.7	21.9	32	28	30	30
Ammoniacal-N	mg N/L	1.58	1.65	1.2	1.0	1.6	1.2	1.3	1.5
Barium	mg/L	-	-	-	-	-	-	1.9	1.2
Lithium	mg/L	-	-	-	-	-	-	1.4	1.5
Strontium	mg/L	-	-	-	-	-	-	4.7	4.7
Iron	mg/L	1.53	0.10	0.07	0.11	-	-	0.31	0.14
Manganese	μg/L	513	832	790	808	-	-	340	360
**Cations (total)**									
Iron	mg/L	1.29	0.05	0.46	0.05	0.41	0.13	0.35	0.17
Manganese	μg/L	531	740	799	800	530	780	340	360
